# Simulation of dosimetry impact of 4DCT uncertainty in 4D dose calculation for lung SBRT

**DOI:** 10.1186/s13014-018-1191-y

**Published:** 2019-01-08

**Authors:** Gang Liu, Fala Hu, Xuanfeng Ding, Xiaoqiang Li, Qihong Shao, Yuenan Wang, Jing Yang, Hong Quan

**Affiliations:** 1 Key Laboratory of Artificial Micro- and Nano- structures of Ministry of Education and Center for Electronic Microscopy, School of Physics and Technology, Wuhan University, Wuhan, 430072 China; 2 Cancer Center, Union Hospital, Tongji Medical College, Huazhong University of Science and Technology, Wuhan, 430022 China; 3 School of Mathematics and Statistics, Wuhan University, Wuhan, 430072 China; 4 Proton Therapy Center Beaumont Health, Royal Oak, MI 48074 USA; 5 Wuhan Zhongyuan Electronics Group Co. LTD, Wuhan, 430205 China; 6 Cancer Hospital Chinese Academy of Medical Sciences, Shenzhen Center, Shenzhen, 518000 China

## Abstract

**Background:**

Due to the heterogeneity of patient’s individual respiratory motion pattern in lung stereotactic body radiotherapy (SBRT), treatment planning dose assessment using a traditional four-dimensional computed tomography (4DCT_traditional) images based on a uniform breathing curve may not represent the true treatment dose delivered to the patient. The purpose of this study was to evaluate the accumulated dose discrepancy between based on the 4DCT_traditional and true 4DCT (4DCT_true) that incorporated with the patient’s real entire breathing motion. The study also explored a novel 4D robust planning strategy to compensate for such heterogeneity respiratory motion uncertainties.

**Methods:**

Simulated and measured patient specific breathing curves were used to generate 4D targets motion CT images. Volumetric-modulated arc therapy (VMAT) was planned using two arcs. Accumulated dose was obtained by recalculating the plan dose on each individual phase image and then deformed the dose from each phase image to the reference image. The “4 D dose” (*D*^4*D*^) and “true dose” (D^true^) were the accumulated dose based on the 4DCT_traditional and 4DCT_true respectively. The average worse case dose discrepancy ($\overline{\Delta D}$) between *D*^4*D*^ and D^true^ in all treatment fraction was calculated to evaluate dosimetric /planning parameters and correlate them with the heterogeneity of respiratory-induced motion patterns. A novel 4D robust optimization strategy for VMAT (4D Ro-VMAT) based on the probability density function(pdf) of breathing curve was proposed to improve the target coverage in the presence of heterogeneity respiratory motion. The data were assessed with a paired t-tests.

**Results:**

With increasing breathing amplitude from 5 to 20 mm, target $\overline{\Delta {D}_{99}}$, $\overline{\Delta {D}_{95}}$ increased from 1.59,1.39 to 10.15%,8.66% respectively. When the standard deviation of breathing amplitude increased from 15 to 35% of the mean amplitude, $\overline{\Delta {D}_{99}}$, $\overline{\Delta {D}_{95}}$ increased from 4.06,3.48 to 10.25%,6.63% respectively. The 4D Ro-VMAT plan significantly improve the target dose compared to VMAT plan.

**Conclusion:**

When the breathing curve amplitude is more than 10 mm and standard deviation of amplitude is higher than 25% of mean amplitude, special care is needed to choose an appropriated dose accumulation approach to evaluate lung SBRT plan target coverage robustness. The proposed 4D Ro_VMAT strategy based on the pdf of patient specific breathing curve could effectively compensate such uncertainties.

## Background

Stereotactic body radiotherapy (SBRT) has demonstrated a significant improvement in local tumor control and overall survival of early-stage lung cancer patients [1–4]. However, dose uncertainty may happen due to the substantial respiratory-induced geometric changes. Currently, the most popular method to compensate for such respiratory-induced target motion in treatment planning is to use four-dimensional computed tomography (4DCT) images with an internal target volume (ITV) design [5]. The patient is commonly in a free-breathing status during the 4DCT simulation. However, the tumor positions manifested on 4DCT images are most likely within a single breathing cycle which is assuming a uniform breathing pattern [6, 7].

However, for lung cancer patients, breathing curve is very likely irregular, the tumor positions shown on the 4DCT images used for treatment planning under estimated the actual tumor motion positions [8, 9]. The traditional 4DCT(4DCT_tradiational) is reconstructed by using 10 phase images to represent a periodic motion which may come from any single breathing cycle and may not fully represent true tumor position and motion [10, 11]. Such uncertainties will introduce variations of the dose in the target particularly in the lung patient with irregular breathing curve/who cannot breathe homogeneously.

Flampouri et al. investigated the dose deviation between planned dose and delivered dose due to respiratory motion and free breathing helical CT artefacts for lung intensity modulated radiotherapy(IMRT) treatments [12]. While the result demonstrated dose difference occurred, delivered dose was only approximated by the deforming and summing of the dose distributions from the ten 4DCT’s phase images [12].

In the presence of irregular breathing curve, it may not be practical achievable to acquire and reconstruct a true and artifact-free 4DCT due to the limited information and image acquisition technique [13]. In this study, we proposed a novel method to generate a true 4DCT(4DCT_true) using a patient’s digital phantom incorporating the irregular patient specific respiratory motion. In order to estimate the delivered dose and evaluate the plan robustness for lung SBRT, clinically a 4D dose accumulation method based on a deformation algorithm and workflow was introduced by Guckenberger et al. in 2006 [14]. However, limited 4DCT_traditional reconstruction, a true dose is difficult to acquire [15]. In order to overcome the limitation of image acquisition on 4DCT patient data, James et al. introduced the digital phantom concept simulating the irregular tumor motion and reconstructed a true dose accumulation [15]. However, the dose discrepancy between the “4D dose” (D^4D^) calculated using 4DCT _traditional and the “true dose” (D^true^) calculated based on 4DCT_true is never studied.

To our best of knowledge, it is very first comprehensive study to investigate the dose discrepancy between the D^4D^ and D^true^ simulating different tumor size, different breathing pattern including amplitude, breathing cycle and heterogeneity of the breathing pattern using simulated a digital tumor phantom. Then three real patients’ breathing curve were used to validate this model result. In addition, we proposed a novel probability density function (pdf) of 4DCT based robust optimization strategy for lung SBRT to improve the target coverage in the presence of such irregular breathing pattern.

## Methods

### Digital lung cancer phantom

**Fig. 1:**
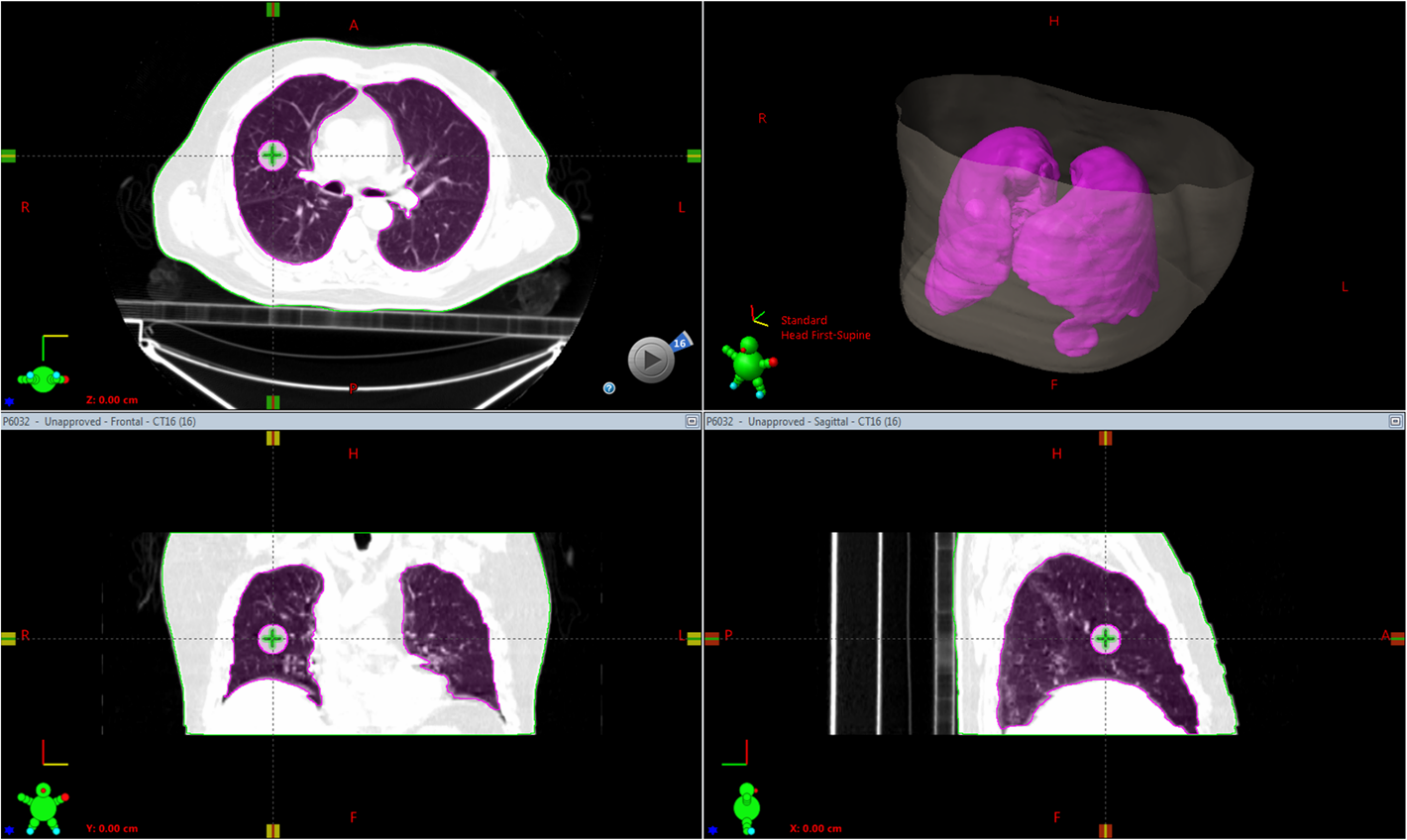
Example of a digital lung cancer phantom. The pink contour represents the gross target volume (GTV)

A digital lung cancer phantom simulation was created to overcome the limited number of phase images and tumor motion positions in the 4DCT_tradition. The tumor motion occurs most significantly along the superior inferior (SI) direction in lung cancer patients, with a peak-peak amplitude range from 0 to 2 cm (95% cases) in most cases but with exceptional amplitude about 3 cm (2% cases) was also observed [16, 17]. This digital phantom was created using a ten-phase 4DCT images with a diaphragm motion of 3 cm from a clinical lung cancer patient. The original phase image has dimensions of 512 × 512 × 80 with a pixel size of 0.098 × 0.098 cm and slice thickness of 0.25 cm. This image was converted to a 500 × 500 × 200 dimension with a 0.1 cm × 0.1 cm × 0.1 cm voxel resolution using tri-linear interpolation. Then the 4DCT images were sorted according to the diaphragm position in right lung and resulted that 0% phase represented diaphragm in valley position, while 90% phase represented peak position, 50% phase is the neutral position. A digital sphere (tumor) with an assigned physical density of 1.0 g/cc, which was approximate to the average density of tumor in lung [18], was inserted into the middle of the right lung on the 50% phase image as shown in Fig. 1. The sphere center was used as the reference coordinate center with each patient dimension, namely, right left (RL), posterior anterior (PA) and SI. An additional 30 sample images were created by inserting the sphere in each of the 10-phase 4D images at the position of RL = PA = 0, with SI = − 15 to 15 mm with a 1-mm increment. The corresponding phase image was selected as following:

$$ {\displaystyle \begin{array}{c}P=\left[\left(i+15\right)/3\right],i=\hbox{-} 15,\hbox{-} 14,\dots 14,\\ {}P=9,i=15,\end{array}} $$

where *i* represents the sphere position in SI direction, [] represents taking the integer portion, *P* represents *P* × 10% phase.

In this study, three different sphere diameters (2, 3 and 4 cm) were used to investigate the effect of the target volume. Therefore, a total of 93 digital sample images were created to mimic the tumors with different volumes at different positions along the SI direction. These sample images were matched with the corresponding breathing phases and were used to mimic variations of the tumor motion pattern as well as real patient’s heterogeneous irregular breathing motion.

### Tumor motion simulation

Tumor heterogeneous breathing motion in the SI direction was simulated using the standard motion function with two random parameters [19],

1 $$ Z(t)=A\times \left({\mathit{\cos}}^4\left(\pi \times \frac{t}{P}\right)-1/2\right) $$

where *Z* is the tumor displacement (unit: mm). The parameter *A* represents the motion amplitude, which is a random variable with a Gaussian distribution *N*(*μ*_*A*_, *σ*_*A*_), where *δ*_*A*_ = *n* ⋅ *u*_*A*_, in which *n* is a determined proportionality coefficient. *P* represents the duty cycle of one breathing cycle, which is also a random variable with a Gaussian distribution *N*(*μ*_*P*_, *σ*_*P*_), where *δ*_*P*_ = *m* ⋅ *u*_*p*_, in which *m* is also a determined proportionality coefficient. Each heterogeneous breathing motion curve in this study comprised several respiratory cycles. Each respiratory cycle within a session varied by changing the peak-to-peak amplitude (*A*) and duty cycle (*P*) sampled from a random generator utilizing the corresponding Gaussian distribution described above. A different number of seeds in the random generator was selected to simulate the respiratory motion among sessions.

In the present study, 4DCT scanning lasted approximately 100 s in a session, which was about the average time of breathing motion recorded during 4DCT scanning in our institution. The simulated respiratory motion image was created using the phase images that reflected the simulated heterogeneity of breathing pattern. Ten different respiratory curves were generated by combining different mean amplitudes, standard deviations of the amplitude, mean period cycles, and standard deviations of the period cycle. The entire combination of simulated breathing pattern parameter is listed in Table 1. Each combination was simulated 10 times, and 100 breathing curves were simulated in total.

**Table 1:** Combination of simulation for the breathing curves

No.	Mean excursion (mm)	Standard deviation of excursion/mean excursion	Mean period (s)	Standard deviation of period/mean period
1	5	0.25	4	0.2
2	10	0.25	4	0.2
3	15	0.25	4	0.2
4	20	0.25	4	0.2
5	15	0.15	4	0.2
6	15	0.35	4	0.2
7	15	0.25	3	0.2
8	15	0.25	5	0.2
9	15	0.25	4	0.1
10	15	0.25	4	0.3

### Tumor motion in actual patient

Three patient’s breathing curves were used for validation purpose. 1.5-mm-diameter gold fiducial markers was implanted into the lungs of three early-stage lung cancer patients. During SBRT treatment, stereoscopic X-ray fluoroscopy images of the gold fiducial markers at a frequency of 30 Hz were acquired over multiple days to determine the marker positions at a 1-mm spatial accuracy [20]. The tumor motion data in the SI direction of three patients with five fractions were acquired during treatment.

To reduce the influence of noise on the observed data and easily acquire peaks and valleys, a low-pass filter that passes signals with a frequency lower than a certain cutoff frequency and attenuates signals with frequencies higher than the cutoff frequency was applied to process real tumor motion data. The exact frequency response of the filter depends on the filter design using fdatool in Matlab (version R2015a, Mathworks). Each actual breathing curve last more than 300 s, but only the previous 100 s were adopted in the present simulation after filtering. The respiratory motion image in actual patient was generated using the phase images that reflect the heterogeneity breathing pattern of the corresponding patient.

### 4DCT reconstruction and treatment planning

The respiratory motion images were generated using the phase images from the specific breathing curve. For 4DCT_traditional reconstruction and planning purpose, four homogenous respiratory motion curves with amplitude A = 5,10,15,20 mm and duty cycle *P* = 4 s were generated using formula (1). The 4DCT_traditional images were created utilizing phase based sorting. The detailed selection of phase images was described as follows [21]: the displacement *Z* was denoted as a function of time *Z(t)* in present study. The time stamps at the peaks and valleys of the respiratory curves are identified. A neighboring peak and valley points represent a cycle, which will be divided into 10 equal time intervals. The symbol *t*^*i*^_*S*_, *t*^*i*^_*E*_ are the starting and ending time of phase *i* then the sampling point will be

2 $$ {P}_i=Z\left(\frac{{t^i}_S+{t^i}_E}{2}\right) $$

The average CT (AVG CT) image was generated by averaging the voxel intensities of all phase image and the ITV was created by adding all phase gross target volume (GTV). To study the dosimetric impact of tumor motion amplitude, four SBRT plans were generated based on the 4DCT_traditional with breathing amplitude from 5 to 20 mm.

A Varian Trilogy (Varian medical system, USA) equipped with the Millennium™ Multi-Leaf Collimator (MLC) was used in this treatment planning study. The VMAT plan with 6MV photon were utilized with 180°–30° clockwise and 30°–180° counterclockwise aimed at ITV center on each AVG CT, where the collimator angles were 350°/10°. And the treatment prescription dose was 60 Gy to the ITV in 5 fractions. The treatment plan achieved a minimal ITV dose of 60 Gy. To investigate the effect of dose gradient on ITV, VMAT plans with ITV surrounding a 70, 80 and 90% isodose line (the proportion between prescription dose and maximum dose in ITV) on the AVG CT generated by static amplitude with 15 mm and target size as 3 cm diameter were also designed. Additionally, to investigate the effect of variations in target size [22], a series of VMAT plans were generated using different target diameters of 2, 3 and 4 cm. The detail of plan was list in Table 2. Phase image with tumor position of SI = 0 mm was set as the reference. All plans were created using inverse planning approach with a collapsed cone (CC) dose calculation algorithm in the Raystation version 6.0 Treatment Planning System (TPS) (Raysearch, Sweden). Dose grid was 0.1 × 0.1 × 0.1 cm^3^.

**Table 2:** The detail of plan

No.	Amplitude (mm)	Duty cycle (s)	Target diameter (cm)	Prescription dose level
1	5	4	3	80%
2	10	4	3	80%
3	15	4	2	80%
4	15	4	3	80%
5	15	4	4	80%
6	15	4	3	70%
7	15	4	3	90%
8	20	4	3	80%
9	10	4	3	80%

### 4D robust optimization based on the pdf strategy

**Fig. 2:**
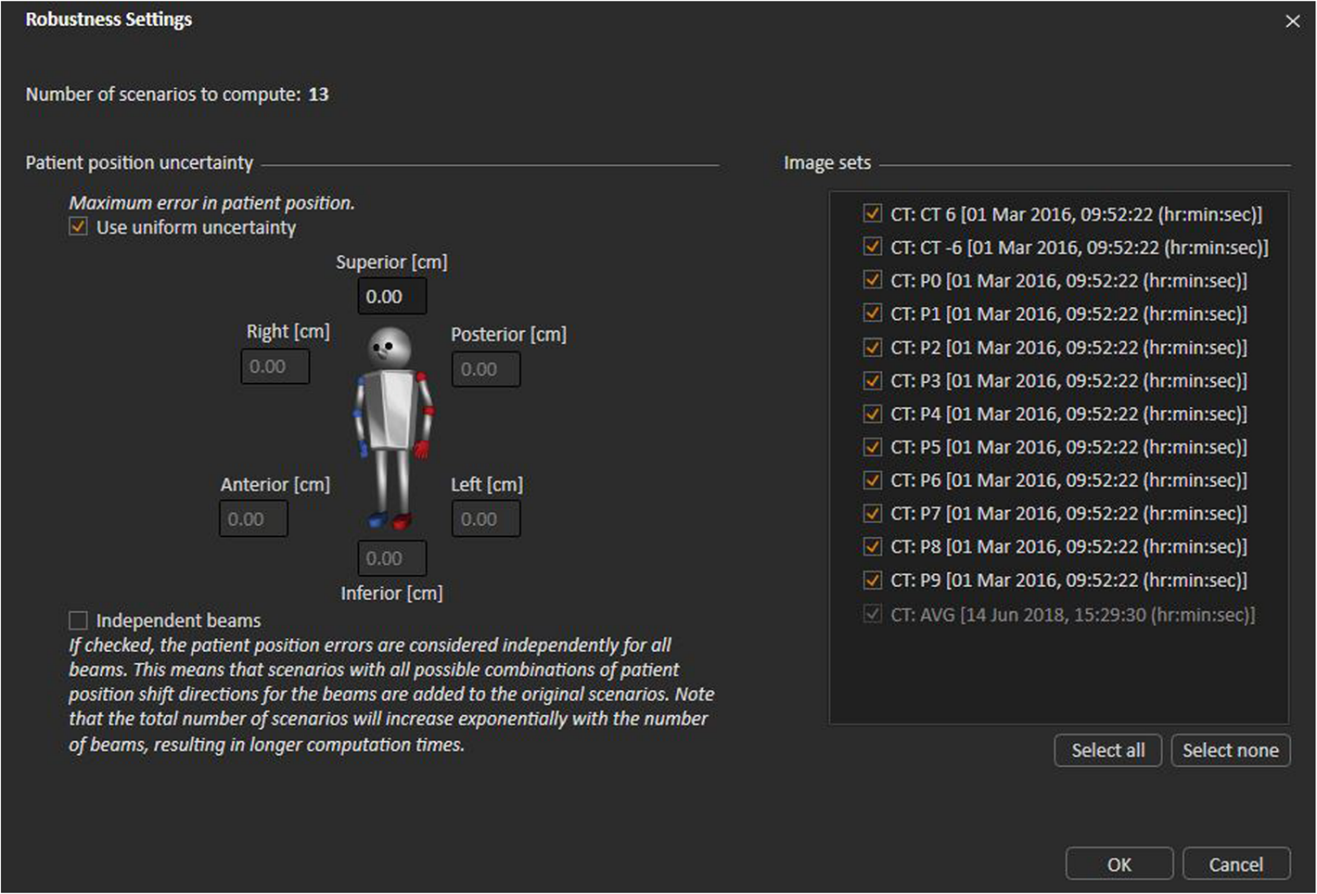
The robustness setting

Robust optimization was initially introduced for intensity-modulated proton therapy (IMPT) to ensure adequate target dose coverage to account for position and proton range uncertainties [23]. It has been available for x-ray beam in radiation therapy treatment planning system recently [24]. A 4D robust optimization method was introduced by Liu et al. to further reduce the impact of uncertainties including intra-fraction organ motion and shape changing to treat lung cancer [25]. The 4D robust optimization plans were constructed by optimizing the GTV dose on ten phases of the 4DCT_traditional. In order to compensate the heterogeneity respiratory motion, a novel 4D robust optimization strategy based on the probability of breathing curve is proposed in this study by adding additional phase image incorporated into the 4D robust optimization. More specifically, based on the 4DCT_traditional, additional phase images was added to cover 80% of tumor position probability during the heterogeneous respiratory motion or 80% of probability density function. For example, for the patient #2 has average amplitude about 10 mm, the 4DCT_traditional only including ten phase images to cover 10 mm range in SI direction from SI = − 5 to 5 mm. In pdf based Ro_VMAT planning strategy, in order to achieve 80% tumor probability position coverage, phase images with SI = − 6, 6 mm were added into the 4D Ro_VMAT planning optimization. The implement of 4D robust optimization was provided in Raystation version 6.0, Fig. 2 displayed the robustness setting as an example. This strategy was validated on three actual patients.

### Treatment dose construction

In order to calculate the accumulated target dose, treatment plans were first recalculated on each phase images. Then the recalculated dose on each phase images were deformed and accumulated into the reference phase image. Phase image with tumor position of SI = 0 mm was set as the reference image in this study. Therefore, accumulated dose construction for each sub-volume, *e*, in the tumor can be written as [26].

3 $$ D(e)=\frac{1}{I}\sum \limits_{i=1}^Id\left({x}_i(e)\right) $$

where *x*_*i*_*(e)* indicates the tumor sub-volume position of a sample image, with the index *i* representing the *i-*th target motion/image sample. *d (x*_*i*_*(e))* represents the point dose at the tumor sub-volume position, *x*_*i*_*(e)*, calculated using a treatment plan based on the sample image. *D*^4*D*^ indicates the accumulated dose calculated using the sample images produced using 4DCT_traditional (10 phase images were used) while *D*^*true*^ indicates a true accumulated dose based on the whole sample images throughout the heterogeneity breathing cycle. *I* is the total number of phase images, set to 10 for *D*^4*D*^ and the total number of phase images *D*^*true*^ calculations (~ 250 phase images in 4DCT_true), respectively. The treatment dose construction considered the tumor sub-volume displacements, but it did not consider the MLC interplay effect.

### Evaluation

*D*_*x*_ was defined as largest dose level percentage covering *x*% volume of the target. *D*^*true*^_*x,j*_ was defined as the true cumulative dose for the *j*-th curve that covers *x*% of the target volume, while *D*^4*D*^_*x*, *j*, *k*_ was defined as the cumulative dose for the *k*-periodic segment of the *j*-th curve that covers *x*% of the target volume.

The relative target accumulated dose deviation on average in the *j*-th curve for the worst case was define as:

4 $$ \overline{\Delta {D}_x}=\sum \limits_{j=1}^J\frac{\max \left\{|{D}^{4D}{{{}_x}_{,}}_{j,k}-{{D^{true}}_{x,}}_j\Big\Vert k=1,\dots, {K}_j\right\}}{J\times {{D^{true}}_{x,}}_j}\times 100\% $$

where *J* is the simulation times or fractions and *J* = 10 in tumor motion simulation, while *J* = 5 from actual patient treatment fractions, and *K*_*j*_ is the periodic amount included in the *j*-th curve. Linear regression was used to fit the $\overline{\Delta {D}_{99}}$ and $\overline{\Delta {D}_{95}}$ relationship between cumulative dose deviation and the variance of the respiratory motion pattern.

The data were assessed with a paired t-tests (non-parametric Wilcoxon signed rank test) using SPSS 19.0 software (International Business Machines, Armonk, New York), and *p* values equal to or less than 0.05 were considered statistically significant.

## Results

### Digital lung cancer phantom simulation and dose accumulation

**Fig. 3:**
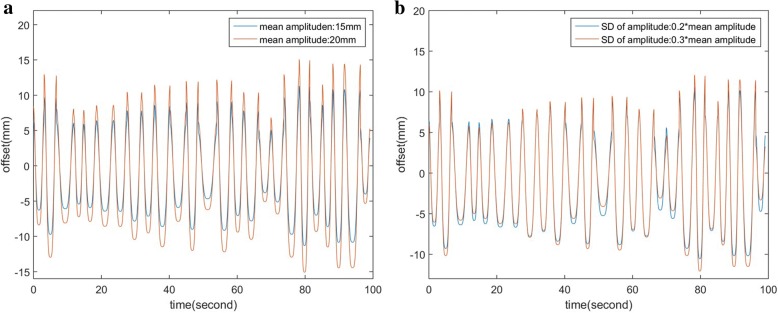
Two different simulated digital phantom respiratory motion with **a** different mean amplitudes and **b** different standard deviations of the amplitude

The heterogeneous motion pattern mimicking real patient breathing curve was simulated, an example in Fig. 3a and b displayed the irregular respiratory motion simulated with different mean amplitude and different standard deviation of amplitude respectively.

**Fig. 4:**
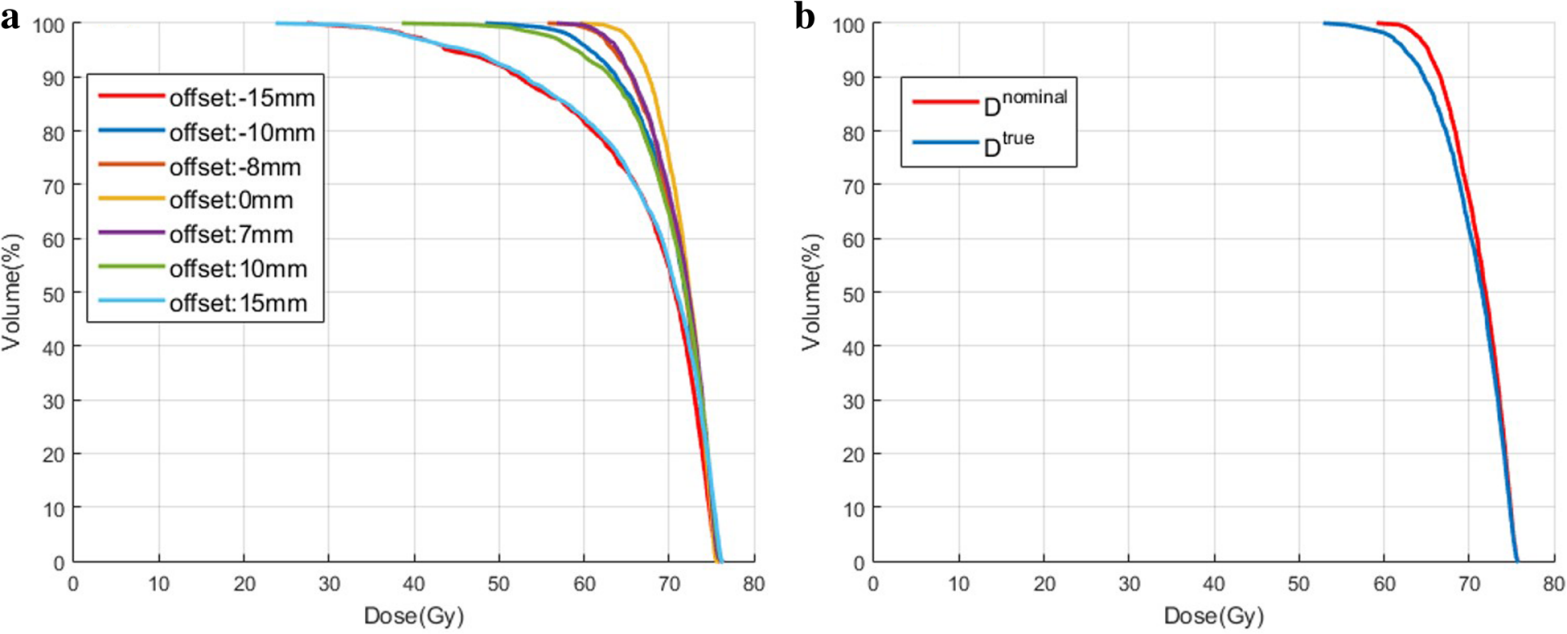
**a** An example for dose volume histograms (DVHs) of the target with different offset values. **b** An example of cumulative dose DVH for D^nomial^ and *D*^*true*^. Where D^nomial^ represents the accumulated dose based on the 4DCT_traditional which was used for planning, D^true^ represents the accumulated dose incorpated heterogeneity breathing pattern with mean amplitude 15 mm, standard deviation of amplitude 3.75 mm, mean period 4 s and standard deviation of period 0.8 s

As we expected, target dose coverage degraded as the tumor position exceed the planning ITV volume due to the heterogeneity of breathing pattern. For example, Fig. 4a, shows the target dose volume histograms (DVHs) for with different offset based on the VMAT plan with ITV 15 mm. The results show that the dose degradation was sensitive to the offset from the reference, but the chance of exceeds the 4DCT_traditional average amplitude may varies depending on each specific patient. Figure 4b shows accumulated dose on target for D^nominal^ and D^true^. Where D^nomial^ represents the accumulated dose based on the 4DCT_traditional which was used for planning with a uniform breathing pattern, D^true^ represents the accumulated dose incorporated heterogeneity breathing pattern with mean amplitude 15 mm, standard deviation of amplitude 3.75 mm, mean period 4 s and standard deviation of period 0.8 s. There was a slightly dose degradation on target due to heterogeneous respiratory motion.

**Fig. 5:**
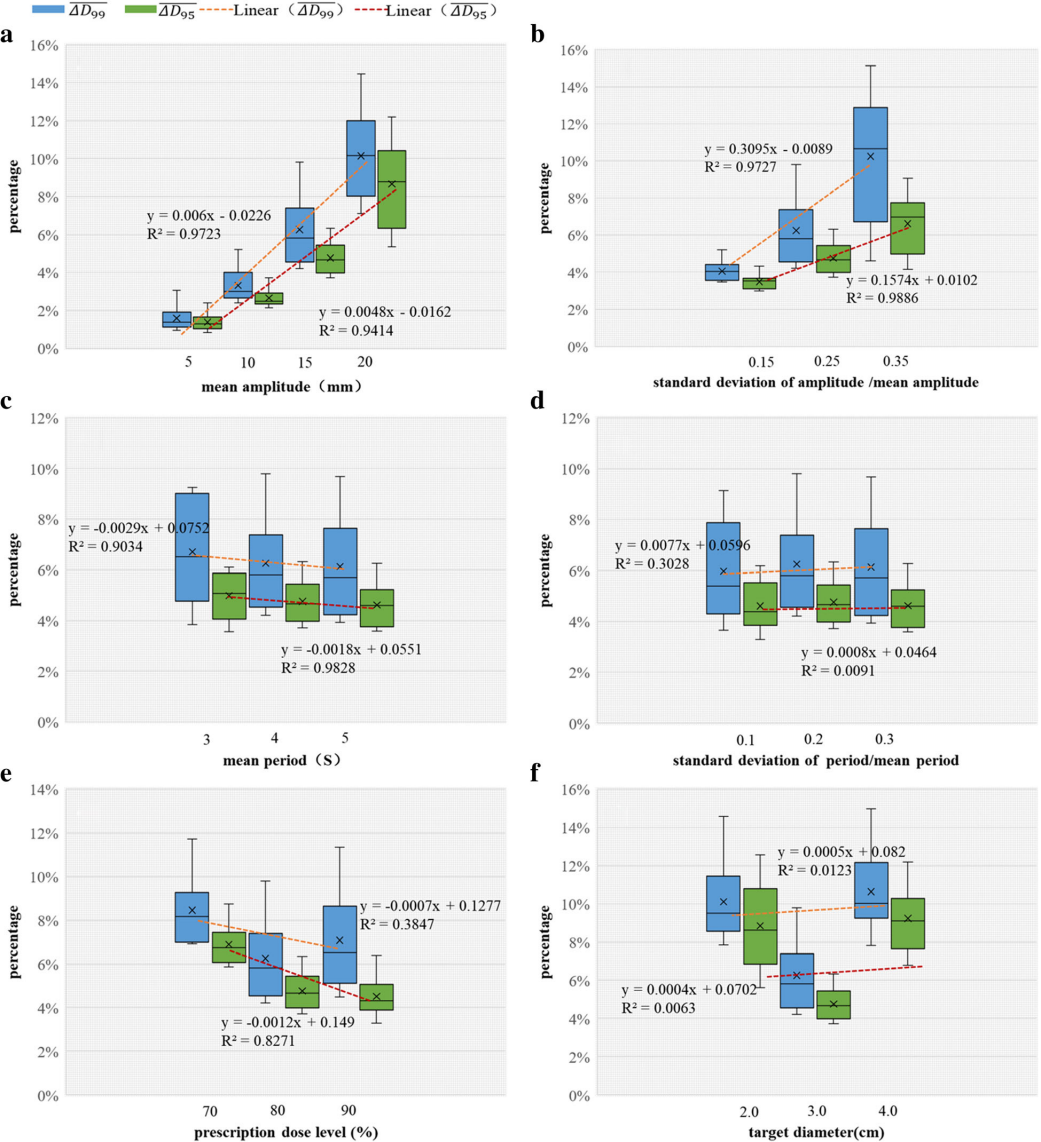
$\overline{\Delta {D}_{99}}$ ($\overline{\Delta {D}_{95}}$) varies with the **a** mean amplitude, **b** standard deviation of the amplitude, **c** mean period, **d** standard deviation of the period, **e** prescription dose level, and **f** target diameter. The static conditions are: **a** standard deviation of amplitude/mean amplitude: 0.25, mean period: 4 s, standard deviation of the period/mean period: 0.2, prescription dose level: 80% and target diameter: 3 cm; **b** mean amplitude: 15 mm, mean period: 4 s, standard deviation of the period/mean period: 0.2, prescription dose level: 80% and target diameter: 3 cm; **c** mean amplitude: 15 mm, standard deviation of the amplitude/mean amplitude: 0.25, mean period: 4 s, standard deviation of the period/mean period: 0.2, prescription dose level: 80% and target diameter: 3 cm; **d** mean amplitude: 15 mm, standard deviation of the amplitude/mean amplitude: 0.25, mean period: 4 s, prescription dose level: 80% and target diameter: 3 cm; **e** mean amplitude: 15 mm, standard deviation of the amplitude/mean amplitude: 0.25, mean period: 4 s, standard deviation of the period/mean period: 0.2, target diameter: 3 cm; **f** mean amplitude: 15 mm, standard deviation of the amplitude/mean amplitude: 0.25, mean period: 4 s, standard deviation of the period/mean period: 0.2, and prescription dose level: 80%

The study found that $\overline{\Delta {D}_{99}}$ and $\overline{\Delta {D}_{95}}$ has a linear relationship with the average amplitude and the standard deviation of amplitude (Fig. 5a and b). $\overline{\Delta {D}_{99}}$ increased from 1.59 to 10.15% and $\overline{\Delta {D}_{95}}$ increased from 1.39 to 8.66% along with an increase in the average amplitude from 5 to 20 mm. When the standard deviation of amplitude varied from 0.15 to 0.35 times the mean amplitude, $\overline{\Delta {D}_{99}}$, $\overline{\Delta {D}_{95}}$ varied in a range from 4.06 to 10.25%, 3.48 to 6.63%, respectively.

The study also found that $\overline{\Delta {D}_{99}}$ and $\overline{\Delta {D}_{95}}$ has a weak correlation with the period time as well as standard deviation of the period time. $\overline{\Delta {D}_{99}}$, $\overline{\Delta {D}_{95}}$ decreased from 6.71, 4.98 to 6.13%, 4.62%, while the standard deviation of the period varied from 10 to 30% of the average period, 5.97% %<$\overline{\Delta {D}_{99}}$< 6.25%,4.60% %<$\overline{\Delta {D}_{95}}$< 4.76% .$\overline{\Delta {D}_{99}}$, $\overline{\Delta {D}_{95}}$ varied along the average and standard deviation of the period as displayed in Fig. 5c and d, respectively.

The relationship between $\overline{\Delta {D}_{99}}$, $\overline{\Delta {D}_{95}}$ and prescription dose level and target volume are shown in Fig. 5e and f, respectively. When the prescription isodose level varied from 70 to 90%, all $\overline{\Delta {D}_{99}}$ values were less than 8.45% and greater than 6.25%, all $\overline{\Delta {D}_{95}}$ were less than 6.89% and greater than 4.51%. When the tumor diameter varied from 2 to 4 cm, $\overline{\Delta {D}_{99}}$ was 10.1%, 6.25 and 10.63%, $\overline{\Delta {D}_{95}}$ was 8.84, 4.76 and 9.23%. These results indicated that there is no strong correlation between the dose deviation and the prescription isodose line (dose gradient) or the target volume.

### Tumor motion in actual patient and dose accumulation

**Fig. 6:**
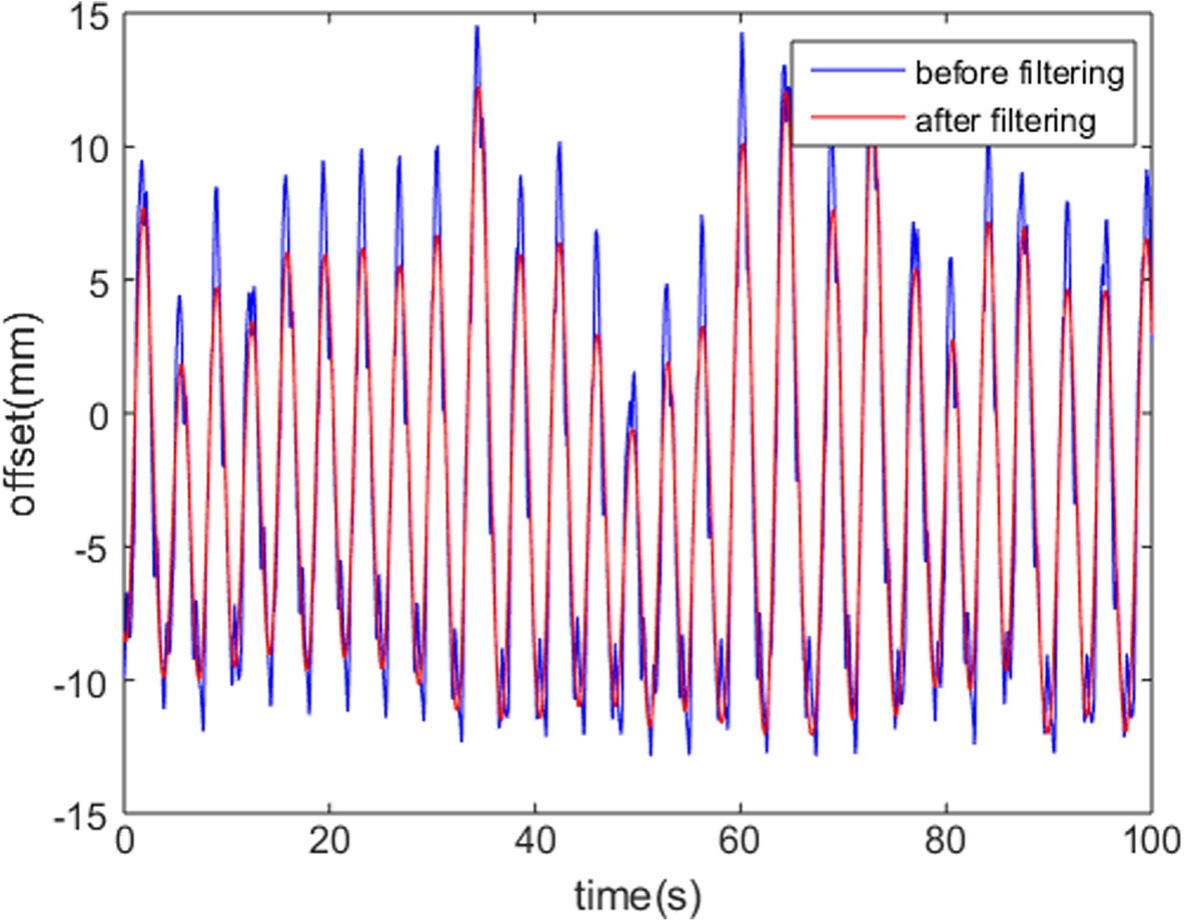
An example of a patient #3 respiratory motion in the SI direction

In order to eliminate noise, filtering is used to smooth the actual patient motion curve. The jagged blue line represents the real tumor motion, while the smooth red line was generated using a digital low-pass filter. Figure 6 shows the respiratory motion in the SI direction for patient #3 as an example. The ranges of the mean amplitude and the standard deviation of amplitude for patient #3 are greater than those for patient #1 and patient #2. The characteristics of the respiratory motion for each patient are described in detail in Table 3.

**Table 3:** Characteristics of the respiratory motion in three patients

No.	Fraction	Mean excursion (mm)	Standard deviation of excursion/mean excursion	Mean period (s)	Standard deviation of period/mean period
1	1	10.11	0.12	4.85	0.12
	2	10.32	0.09	4.65	0.09
	3	10.14	0.12	4.95	0.08
	4	9.88	0.11	5.15	0.09
	5	8.59	0.09	5.05	0.12
2	1	10.21	0.23	3.78	0.05
	2	9.92	0.20	3.81	0.09
	3	10.53	0.23	3.85	0.09
	4	10.62	0.25	3.39	0.15
	5	10.05	0.22	3.78	0.05
3	1	15.28	0.23	2.71	0.08
	2	14.36	0.20	2.79	0.08
	3	16.21	0.21	3.18	0.11
	4	15.88	0.23	3.11	0.10
	5	15.20	0.22	3.06	0.10

$\overline{\Delta {D}_{99}}$, $\overline{\Delta {D}_{95}}$ for the three patients were 2.15,1.98% for patient #1, 3.48%, 2.68% for patient #2, and 6.63%, 4.74% for patient #3 based on the simulation. D^4D^ evaluation approach may not be appropriate in the presence of the accumulated dose with heterogeneous breathing motion. Although a complicated respiratory pattern was calculated from the actual patient, the results are consistent with those from the phantom study.

#### 4D Ro-VMAT outcome

**Fig. 7:**
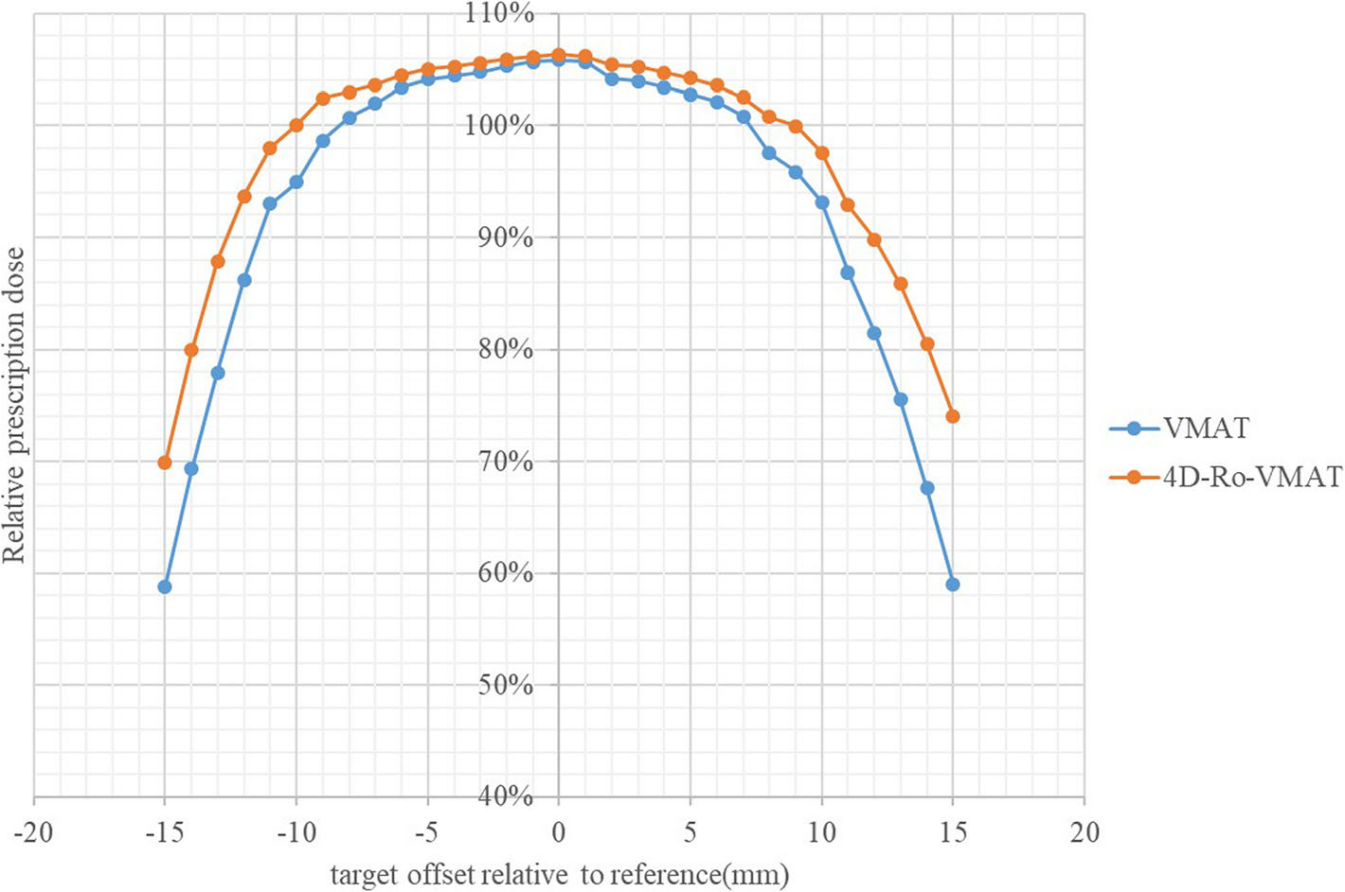
An example of the phase target dose D_99_ vs tumor position comparison between VMAT and 4D-Ro-VMAT

Due to additional phase images was included in the pdf based 4D Ro_VMAT planning strategy, the target dose coverage was improved compared to VMAT plan. The target dose D_99_ vs tumor position in each phase image was displayed in Fig. 7 from patient #3. The pdf based 4D-Ro-VMAT could effectively compensate the target dose degradation which was out of ITV boundary. When the respiratory pattern is severely irregular, 4D-Ro-VMAT shows a significant improvement in those in these extreme target positions although the chance of such displacement is rare or very lightly weighted. Compared to the VMAT plan, the target accumulated dose ${D}_{99}^{true}$ significantly increased 1.9% (*p* < 0.01) and 2.1% (p < 0.01) on average throughout the 5 fractions in the 4D Ro-VMAT plan for patient #2 and #3 respectively, the 4D Ro-VMAT plan significantly improve the accumulated dose coverage. However, when the mean amplitude is less than 10 mm or the amplitude standard deviation is less than 1 mm for patient #1, the accumulated dose ${D}_{99}^{true}$ increases 0.1%(*p* = 0.21) on average during 5 fractions, 4D Ro-VMAT may not be necessary.

## Discussion

The study found that the heterogeneity breathing pattern with a greater mean amplitude or the standard deviation of the amplitude led towards a larger variations of target motion and position (Fig. 3a and b). Such phenomena resulted in a significant deviation of the accumulated dose between D^4D^ and D^true^ evaluation approaches, especially when the average breath amplitude is larger than 10 mm or standard deviation of amplitude is larger than 25% of mean amplitude. In order to minimize the breathing curve standard deviation, Blomgren et al. have reported that a passive pressure technique can limit the movement of the diaphragm-by-diaphragm pressure plate, thereby reducing the amplitude of the tumor motion [27]. In addition, respiratory motion is a semi-autonomous and irregular motion, patient breathing training may be helpful to improve the reproducibility of the respiratory amplitude and frequency. Shallow breathing control or high frequency breathing technique can be utilized as well [28]. Therefore, the use of the above techniques can reduce the uncertainty caused by breathing motion thus achieve to improve the accuracy of the accumulated dose calculated with 4DCT_traditionl.

The study found that the accumulated dose discrepancy slightly varies along with the variation of mean and the standard deviation of the period cycle. However, it should be noted that such phenomena might varies if treatment time delivery and tumor motion interplay effect is considered. The study also showed that the target absolute volume doesn’t affect the accumulated dose evaluation approach using D^4D^ or D^true^. Furthermore, the accumulated dose discrepancy is independent of the prescription isodose line (dose gradient) as well [29], since dose gradient described dose variation along with voxel position variation, while *D*_*x*_ discribed the largest dose level percentage covering *x*% target volume.

This study assuming the real-time tumor position is available for D^true^ evaluation approach. However, in the current clinical practice, 4DCT_traditional simulation may not fully represents true tumor position and motion [11]. In order to achieve the real-time tumor position tracking, Zhuang et al. proposed an accurately method to extract tumor respiratory motion using the cone beam CT (CBCT) projections during VMAT treatment of lung tumor recently [30]. The result demonstrated that the 3D (x, y, z) mean tumor position and 3D trajectory reconstruction are accurate within ±0.5 mm. Such approach could be implemented into clinical practice and provide the real-time target tracking during the virtual simulation. The study demonstrated a practical feasible way to use D^true^ to evaluate the patient specific tumor motion and provide a more accurate treatment plan robustness analysis using D^true^ approach in case of the patient with a potential larger heterogeneity in breathing pattern and amplitude.

To overcome and mitigate the uncertainties a novel planning strategy was introduced in this study combining the 4D robust optimization approach and the probability density function of breathing pattern. Several 4D robust optimization methods were introduced to address this breathing induced issue. For example, the strategy was implemented by incorporating the min-max optimization of the GTV dose on all phases included in the 4DCT_traditioanl [25]. The similar study from Archibald et al. also showed that such 4D robustness optimization approach could provide a greater stability in both maximum (< 3%) and minimum dose variations (< 2%) over all other techniques included planning target volume (PTV) expansions, ITV with and without tissue override [31]. However, these previous studies using the 4D robust optimization was based on the assumption that respiratory motion throughout the treatment maintained the same pattern manifested on the 4DCT_traditional. In the presence of the heterogeneous breathing pattern in some patients, Chan et al. developed a robust optimization approach incorporating with the convolution of motion pdf variation with static dose distribution, which could mitigate breathing uncertainty as well [32]. However, such approach to calculate the accumulated dose using convolution may not represent the dose distribution with motion accurately [33].

In this study, we proposed to use additional phase images to cover 80% of probability density function for 4D robust optimization. The preliminary result demonstrated that it could significantly reduce the dose uncertainty compared to the VMAT plan when the target mean amplitude was greater or equal to 10 mm and the standard deviation of amplitude was greater or equal to 25% of mean amplitude. This study also indicated that when the standard deviation of amplitude was less than 15% of mean amplitude, the 4D-Ro-VMAT approach may not be effective since the discrepancy between D^4D^ and D^true^ is very minimum. But the selection of appropriate additional phase images to spare normal tissue need to be further studied.

Other limitations in the study are that only rigid motion was investigated; thus, non-rigid motions, such as anatomic changes, needs to be included in the future study. The MLC interplay effect is not considered either.

## Conclusions

Traditional D^4D^ approach might not provide a comprehensive dose accumulation when the respiratory-induced target/organ motion has a larger heterogeneous pattern (more than 10 mm amplitude or 25% mean amplitude as standard deviation of amplitude). D^true^ is preferred in these scenarios. The study suggested that the 4D-Ro-VMAT could be a potential planning strategy to mitigate patient’s breathing pattern heterogeneity.

## Abbreviations

$\overline{\Delta D}$: The average worse case dose discrepancy
4D Ro-VMAT: 4D robust optimization strategy for VMAT
4DCT_traditional: Traditional four-dimensional computed tomography
4DCT_true: True 4DCT
AVG CT: Average CT
CBCT: Cone beam CT
CC: Collapsed cone
*D*^4 *D*^: 4D dose
D^true^: True dose
DVH: Dose volume histogram
*D*_*x*_: The largest dose level percentage covering *x*% volume of the target
GTV: Gross target volume
IMPT: Intensity-modulated proton therapy
IMRT: Intensity modulated radiotherapy
ITV: Internal target volume
MLC: Multi-Leaf Collimator
PA: Posterior anterior
pdf: Probability density function
PTV: Planning target volume
RL: Right left
SBRT: Stereotactic body radiotherapy
SI: Superior inferior
TPS: Treatment Planning System
VMAT: Volumetric-modulated arc therapy
